# The Role of Light Kappa and Lambda Chains in Heart Function Assessment in Patients with AL Amyloidosis

**DOI:** 10.3390/jcm10061274

**Published:** 2021-03-18

**Authors:** Emilia Czyżewska, Agnieszka Wiśniewska, Anna Waszczuk-Gajda, Olga Ciepiela

**Affiliations:** 1 Department of Laboratory Medicine, Medical University of Warsaw, Banacha 1a, 02-097 Warsaw, Poland; emilia.czyzewska@wum.edu.pl (E.C.); agnieszka.wisniewska@wum.edu.pl (A.W.); 2 Department of Hematology, Oncology and Internal Diseases, Medical University of Warsaw, Banacha 1a, 02-097 Warsaw, Poland

**Keywords:** primary amyloidosis, cardiac amyloidosis, immunoglobulin free light chain, monoclonal gammopathy

## Abstract

There are reports indicating that myocardial dysfunction in systemic immunoglobulin light chain amyloidosis (AL amyloidosis) stems not only from the amyloid deposit in the organ but also the cardiotoxicity of the amyloid precursor free light chains (FLCs) circulating in the blood. The aim of the study is to analyze the role of sFLC κ and λ in the assessment of heart involvement and the degree of myocardial damage in AL amyloidosis. The study involved 71 patients diagnosed with primary AL amyloidosis. The relationship between sFLC concentrations and cardiac biochemical and echocardiographic parameters was assessed. The median concentrations of N-terminal pro b-type natriuretic peptide(NT-proBNP) and troponin I (TnI) were significantly higher in patients with amyloids formed from monoclonal λ chains compared to patients with monoclonal κ proliferation. In patients with heart involvement by amyloids formed from monoclonal FLC, the study demonstrated a statistically significant positive correlation between the concentration of monoclonal antibody λ chain and TnI (R = 0.688; *p* < 0.05), NT-proBNP (R = 0.449; *p* < 0.05), and the value of diastolic dimension of the interventricular septum (IVS; R = 0.496, *p* < 0.05). The above data indicate that the presence of monoclonal λ chains in patients with AL amyloidosis may be associated with more severe damage to cardiomyocytes and dysfunction of the myocardium.

## 1. Introduction

Light chain amyloidosis (immunoglobulin light chain amyloidosis (AL)) is caused by clonal neoplastic plasma cells or B-cell clones that produce misfolded light chains (κ or λ). Misfolded proteins deposited in tissues result in progressive organ damage [1,2]. AL amyloidosis is a progressive disease without a tangible cause [3]. Lack of symptoms, different clinical features, and diagnostic limitations are common causes of late diagnosis [4]. It should be included in the differential diagnosis of the following five syndromes: nephrotic syndrome in patients who have not been treated for diabetes; cardiomyopathy confirmed by echocardiography, which is not the result of myocardial ischemia; hepatomegaly (with a proper image of its parenchyma assessed in imaging studies) or increased alkaline phosphatase activity; neuropathy with the presence of monoclonal protein (M) in serum; monoclonal gammopathy of undetermined significance (MGUS), accompanied by weight loss, unexplained fatigue, peripheral edema, or paresthesia [3]. Overall survival of untreated patients from the time of diagnosis is only 12 months, and, for treated patients, it is about 2 years [3]. AL diagnosis criteria include (1) presence of organ abnormalities resulting from amyloid deposition (heart, kidney, liver, nervous system, gastrointestinal tract); (2) presence of amyloid confirmed by Kongo red staining in tissue biopsy (adipose tissue, bone marrow) or organ biopsy; (3) evidence that amyloid is composed of light chain immunoglobulins (immunohistochemistry, immunofluorescence, electron microscopy, mass spectrometry); (4) diagnosis of plasma cell dyscrasia (confirming the presence of monoclonal protein in serum and/or in urine or clonal plasma cells in bone marrow, and an abnormal free light chain (FLC) κ/λ ratio- <0.26 or >1.65). Systemic AL recognition requires all four listed criteria to be fulfilled [2,3]. The latest risk stratification, formulated by Kumar et al. in 2012, is based on NT-proBNT, cardiac troponin T (TnT), or troponin I (TnI) concentrations, and the difference between the concentration of involved and uninvolved free light chains (dFLCs), the value of which differs between cancer process (monoclonal, amyloidogenic) and nonmalignant hypergammaglobulinemia [5].

The main unfavorable risk factor for AL is cardiac involvement since 75% of deaths in this group of patients are caused by heart failure or arrhythmias resulting from amyloid fiber deposition [6,7]. In addition, the treatment choice depends on myocardium involvement. Cardiomyopathies are the main death cause in patients with AL. The diagnosis of heart involvement is made too late due to the unspecific nature of the symptoms and the lack of early characteristic changes visible in imaging studies [2,8]. Overall survival, estimated at 1.3 years from diagnosis for patients with amyloid cardiomyopathy, is comparable to the worst prognostic cancer [9]. In contrast, the symptoms of heart failure indicate a fulminant course of the disease and shortened overall survival for untreated patients to 4–6 months [10,11]. From the diagnostic point of view, the attempt to assess the relationship between sFLC concentration, laboratory, and echocardiographic parameters for the evaluation of myocardial status in the AL group of patients seems necessary. Particular consideration of the relationship between the type of monoclonal chain (κ or λ)-forming amyloid and the severity of heart dysfunction would be an attempt to choose a chain as an unfavorable prognostic factor [12].

This study aims to analyze the relationship between the free ĸ and free λ chain serum concentration and myocardial involvement markers. We also aim to evaluate which of the monoclonal chains, ĸ or λ, is associated with greater myocardium damage and could be an unfavorable prognostic factor in AL amyloidosis.

## 2. Materials and Methods

### 2.1. Study Group

The study included patients of the Department of Hematology, Oncology and Internal Diseases of the Medical University of Warsaw with primary AL light chain amyloidosis. In this group of patients, a retrospective analysis of the results obtained in the period between January 2009 and May 2016 was performed both as part of initial diagnostics and during treatment monitoring. The criteria for the diagnosis of primary systemic AL amyloidosis included the following:

- Presence of secondary organ abnormalities resulting from amyloid deposition based on the applicable criteria for organ involvement in AL amyloidosis (Table 1) [13,14];
- Amyloid presence confirmed by Congo Red staining of tissue biopsies (organ biopsy, adipose tissue, bone marrow, gingiva);
- Confirmation that amyloid is made of monoclonal immunoglobulin light chains (immunohistochemistry);
- Confirmation of the presence of plasmocyte dyscrasia (presence of monoclonal protein in the serum and/or urine, an incorrect value of the ratio of free κ and λ light chains in the serum, clonality of plasmocytes in bone marrow).

**Figure 1:**
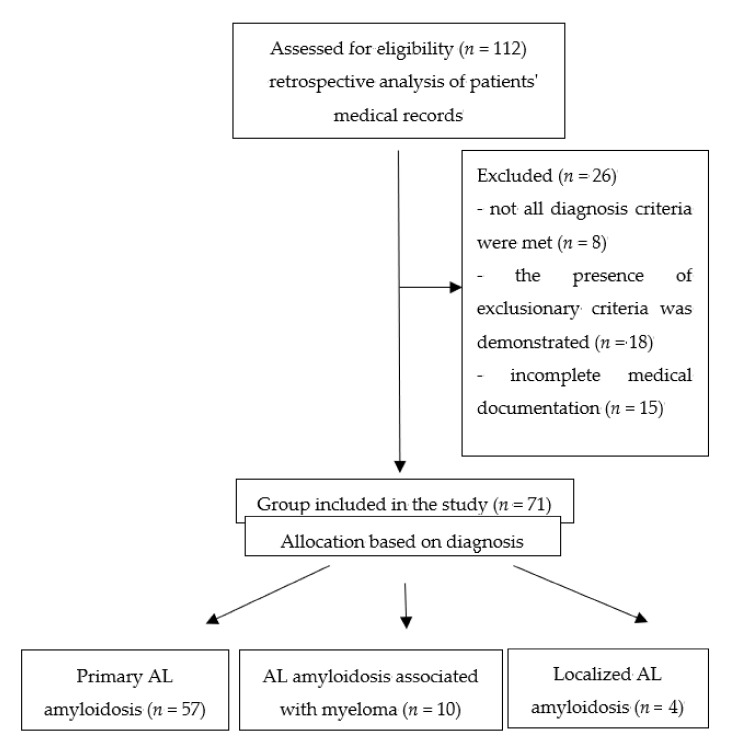
CONSORT diagram—study group selection. AL-Amyloid light-chain

All four of the above criteria had to be met for the diagnosis of primary systemic AL amyloidosis. In localized amyloidosis, the only diagnostic criterion was the presence of amyloids localized in one organ and composed of light chains of immunoglobulins, without the presence of monoclonal proteins in serum and/or urine [2,3]. Exclusion criteria were, among others, history of hypertension, non-amyloid heart disease, valvular disease, atrial fibrillation, hyperthyroidism, Cushing’s syndrome, primary hyperaldosteronism, acute kidney damage, cirrhosis of the liver with ascites, subarachnoid hemorrhage, injuries of the central nervous system, stroke, intake of thyroid hormones, sepsis, and obesity. These data were obtained with the consent of the clinicians from medical records. The study group selection process was presented using the CONSORT diagram (Figure 1). The study group consisted of 71 patients (34 women and 37 men). The mean age at diagnosis or inclusion in the study was 58 ± 11 years. Characteristics of the study group regarding the final diagnosis are presented in Table 2.

Comparative studies included type of monoclonal light chain forming amyloid (ĸ, λ); percentage of plasmocytes with trepanobioptosis at the time of diagnosis; echocardiography parameters, including LV (left ventricular and diastolic dimension), IVS (diastolic ventricular size), PW (left ventricular diastolic wall thickness), RV (right ventricular and diastolic dimension), and EF (left ventricle ejection fraction); laboratory findings, including NT-proBNP, TnI concentrations as parameters assessing cardiac dysfunction and damage, the concentration of free ĸ and λ chains in serum, difference between the concentration of involved and uninvolved free light chain (dFLC), and the free ĸ and λ light chain ratio (ĸ/λ). The number of the analyzed data was not equivalent to the patients’ number. The results were evaluated, including both diagnosis and treatment monitoring. In addition, the commissioned test panels did not cover the compared parameters each time. The study population was a very diverse group in terms of frequency, number, and type of commissioned tests. There were patients monitored every two months for four years, including those who had a one-day stay at the hospital. Laboratory findings and echocardiography were performed in groups based on type of monoclonal amyloid-forming chain (ĸ, λ); FLC ratio (ĸ/λ): <0.26, 0.26–1.65, >1.65; dFLC: <180 and >180 mg/L; clinical stage based on Kumar et al.’s [5] prognostic classification, including TnT ≥ 0.025 ng/mL or TnI ≥ 0.07 ng/mL, NT-proBNP ≥ 1800 pg/mL, and dFLC ≥ 180 mg/dL (Stage I—0 factors, Stage II—1 factor, Stage III—2 factors, Stage IV—3 factors) and diagnosis (AL amyloidosis, AL amyloidosis associated with myeloma, localized AL amyloidosis).

### 2.2. Laboratory Methods

FLC ĸ and λ concentration was measured by the immunoturbidimetric method on Cobas c501 (Roche, Rotkreuz, Switzerland) using Freelite^®^ Human Kappa/Lambda (binding site, Birmingham, UK). Then, the ratios of ĸ/λ FLC in serum (reference range 0.26–1.65) and dFLC were calculated. NT pro-BNT and TnI concentrations were measured using a Dimension EXL analyzer (Siemens Healthcare Diagnostics, Newark, NY, USA) using a one-step sandwich chemiluminescent method based on the Luminescent Oxygen Channeling Immunoassay (LOCI^®^) method. Reference intervals were less than 125 pg/mL for patients aged less than 75 years old and less than 450 pg/mL for patients aged 75 and more years old for NT pro-BNP and 0.000–0.056 ng/mL for TnI. Echocardiography was performed ±2 weeks from the laboratory test (data was based on medical records). The following data from echocardiography were evaluated: left ventricular end-diastolic dimension (LV; reference interval 42–59 mm); diastolic dimension of the interventricular septum (IVS, reference value <12 mm); thickness of the posterior wall of the left ventricle in diastole (PW, reference value <12 mm); right-ventricular end-diastolic dimension (RV, reference value <33 mm); ejection fraction of the left ventricle (EF, reference value >55%).

### 2.3. Statistical Analysis

Results are presented as median and interquartile range (IQR) or absolute numbers and percentages where appropriate. The Kolmogorov–Smirnov test was used to assess the normality of the distributions. In the comparative analysis of the tested material, depending on the type of amyloid-forming monoclonal chain (κ, λ) and dFLC values, the Wilcoxon test was performed.

In the comparative analysis of the tested material, depending on the ratio of FLC κ/λ, clinical stage, and type of organ affected by amyloids, ANOVA by rank was used. Correlation analysis was performed using the Spearman test. In multivariable linear regression, ANOVA was applied with TnI and NT-proBNP as dependable variables. Statistica 12.0 (StatSoft, Tulsa, OK, USA) was used for statistical analysis. *p* < 0.05 was considered significant.

A retrospective study was accordant with the rules of the Bioethical Committee of the Medical University of Warsaw.

## 3. Results

The laboratory results and clinical analysis of subjects with κ and λ amyloids are presented in Table 3.

To assess the difference in the laboratory and clinical results of patients whose FLC ratios (κ/λ) were <0.26, 0.26–1.65, and >1.65, a one-way ANOVA test was performed. There were no differences in age, LV, PW, EF, and RV parameters. However, statistically significant differences were found for plasmocytes percentage, TnI, NTpro-BNP, κ and λ concentrations, dFLC, κ/λ, and IVS parameters (Table 4).

Comparative analysis of study groups with involved and uninvolved free light chains—dFLC <180 and ≥180 mg/L—is presented in Table 5. There were no differences in age, percentage of plasmocytes, κ concentration, κ/λ, LV, RV, and EF values. However, statistically significant differences were found for TnI, NT-proBNP, λ, κ ”i“, λ ”i“, κ ”ui”, λ ”ui” concentrations and IVS and PW parameters.

The study group was divided into four subgroups depending on Kumar et al.’s classification (Stages I–IV). Comparative analysis of the obtained results is presented in Table 6.

Median free light λ “i” concentration in serum was significantly higher in Stage IV vs. Stages I, II, and III. All patients from the Stage IV group have shown the presence of monoclonal λ chains. It was shown that the median IVS values in Stages III and IV were significantly higher than in Stages I and II, and the median of PW and RV values were significantly higher in Stage IV. The median EF value was significantly lower in Stage IV compared to Stages I, II, and III. A comparative analysis, depending on the diagnosis, is presented in Table 7.

The concentrations of TnI and NT-proBNP and the value of PW show significantly lower values in the group of patients with localized AL amyloidosis.

Multivariable linear regression analysis of the group with involvement of the myocardium by amyloids formed from monoclonal λ chains, with TnI and NT-proBNP as dependent variables, was performed.

For TnI (*p* = 0.003), the independent predictor retained in the final regression model was λ. The remaining factors were eliminated (κ, κ/λ, dFLC, eGFR). A λ rise of 1 mg/L caused a rise of TnI concentration of 0.0005 ng/mL (Table 8).

For NT-proBNP (*p* < 0.001), the independent predictors retained in the final regression model were λ, eGFR, and κ. The remaining factors were eliminated (κ/λ, dFLC). A λ rise of 1 mg/L caused a rise of NT-proBNP concentration of 41 pg/mL, while an eGFR decrease of 1 mL/min 1.73 m^2^ caused an increased NT-proBNP concentration of 63 pg/mL. A κ increase of 1 mg/l caused an increase in NT-proBNP of 114 pg/mL (Table 8).

Multivariable linear regression analysis of the group with involvement of the myocardium by amyloids formed from monoclonal κ chains, with TnI and NT-proBNP as dependent variables, was performed. In the group with cardiac involvement where the amyloid is formed by monoclonal κ chains, no significant independent influence of the concentration of free light chains κ and λ, κ/λ, dFLC, and eGFR values on TnI concentrations was observed (*p* > 0.05).

For NT-proBNP (*p* <0.001), the independent predictor retained in the final regression model was λ. The remaining factors were eliminated (κ, κ/λ, dFLC, eGFR). A λ rise of 1 mg/L caused an increase in the NT-proBNP concentration of 204 pg/mL. (Table 9).

In the further analysis a correlation between free light chains parameter and other laboratory and echocariography findings was evaluated for groups with the involvement of the myocardium by amyloid formed of monoclonal λ and κ chains. A strong correlation (r>0.5) was found for TnI and *λ*, TnI and dFLC, as well as for TnI and κ/λ. All results are presented in Table 10.

## 4. Discussion

The heart is, next to the kidneys, an organ in which amyloids during amyloidosis accumulate most frequently. One of the goals of this study is to assess the relationship between the concentration of free κ and λ light chains and markers of myocardial injury and dysfunction—TnI, NTpro-BNP—and echocardiography parameters in patients with AL amyloidosis. An attempt was made to choose the type of monoclonal chain that is associated with greater damage and dysfunction of the heart, which may turn out to be a potential adverse prognostic factor in AL amyloidosis. In the present study, in a group of 71 patients with AL amyloidosis, in 43 (61%), amyloid was formed from monoclonal λ chains, and, in 28 (31%), amyloid was formed from monoclonal κ chains. This confirms the thesis that in patients with AL amyloidosis, clonal plasmocytes are more likely to produce the monoclonal chain λ, in contrast, for plasmocyte myeloma and non-amyloid deposit diseases of monoclonal immunoglobulins, where the presence of a monoclonal κ chain (3:1) is more frequent [4,15,16]. In 51 patients, 72% of organ involvement was in the myocardium, and 41 patients (58%) had amyloid in the kidneys, which is also reflected in the general characteristics of organ involvement in AL amyloidosis [2,5,17,18]. The results of the present study show an elevated median concentration of both TnI and NT-proBNP, as well as an increased median diastolic ventricular size (IVS 13 mm). The increase in the concentration of laboratory cardiac parameters is related to the damage of cardiomyocytes, which is manifested by the increased concentration of TnI and myocardial dysfunction, which is a consequence of pressure and/or volume overload that, in turn, leads to an increase in NT-proBNP. In this study, a comparative analysis, depending on the type of amyloid-forming monoclonal chain, showed that patients with monoclonal λ proliferation had significantly higher median NT-proBNP and TnI concentrations than patients with monoclonal κ chain proliferation. In addition, median dFLC values in patients with monoclonal λ proliferation were significantly lower than in the monoclonal group with a κ chain. Similar relationships were presented in the study by Kumar et al., carried out at the reference center at the Mayo Clinic, in which the relationship between the type of FLC and clinical symptoms in 730 patients with diagnosed AL amyloidosis was analyzed [12]. In the study, 72% of subjects were patients in whom amyloid was formed from λ chains; in 28%, the κ monoclonal chains were an amyloid fiber precursor. The study confirmed a lower median concentration of involved chains and lower dFLC values in the group of patients with monoclonal λ chains compared to monoclonal κ chains. It was suggested that monoclonal λ chains may be more “amyloidogenic” than the κ chains. Min-Ho Lee et al. showed that among 56 AL patients with amyloidosis, λ monoclonal chains were found in 45 of them (80.4%) and κ chains in 11 (19.6%) of them [19]. Similar results were obtained by Huang X et al. and Dispenzierie et al. [20,21]. An important diagnostic parameter is the κ/λ ratio. Our study shows that among patients with a ratio of κ/λ < 0.26, the median TnI concentration was significantly higher than in the group with ratios of >1.65 and 0.26–1.65. The median NT-proBNP concentration also showed the highest values in this group. Similarly, the mean IVS value was significantly higher in the group with κ/λ < 0.26 than in the other group. Laboratory parameters with a confirmed prognostic significance of shorter overall survival are as follows: increased number of plasmocytes in the trepanobiopsy and an increased dFLC value [1,12,22]. Our own study showed that the highest median numbers of plasmocytes and dFLC values were found in patients with a ratio of κ/λ < 0.26.

In the group with dFLC values ≥180 mg/L, considered an unfavorable prognostic factor in patients with AL amyloidosis [12], the median concentration of both TnI and NT-proBNP showed significantly higher values than in the group with dFLC < 180 mg/L. Similarly, the median IVS value in patients with dFLC ≥ 180 mg/L was significantly higher than patients with dFLC < 180 mg/L. Interestingly, in the group with dFLC ≥ 180 mg/L, the median concentration of κ-involved chains was 616.3 mg/L, with the involved λ chains at 289.3 mg/L. Based on these results, it can be suggested that a lower mean concentration of λ monoclonal chains versus κ chains leads to relatively more severe heart dysfunction and myocardial damage. It is problematic, however, to explain the pathomechanism of this dependence itself. This raises the question: are monoclonal λ chains more “amyloidogenic” or does the amyloid formed from them have stronger cardiac tropism and greater cardiotoxicity?

The average concentration of the involved κ chains was significantly higher in Stage II patients compared to Stages I and III. None of the Stage IV patients showed the presence of monoclonal κ chains. The median concentrations of the involved λ chains increased with the severity of the disease (Stages I–IV). The highest median concentration of involved λ chains was found at Stage IV, in which the median total survival was 5.8 months, and the lowest at Stage I, with median survival assessed at 94.1 months. The highest median concentrations of the involved κ chains were at Stage II, with a total median survival of 40.3 months. It can be concluded that the presence of amyloid formed from λ monoclonal chains and the increase in the concentration of its precursors are potentially associated with the shorter survival of patients with AL amyloidosis. The increase in the concentration of monoclonal λ chains was also associated with changes in echocardiographic parameters characteristic for cardiac amyloidosis. The smallest median value of EF% was related to Stage IV, compared to Stages I, II, and III. Moreover, a comparative analysis, depending on the diagnosis, showed that the concentrations of TnI and NT-proBNP and the value of PW showed significantly lower values in patients with localized AL amyloidosis. In this group, circulating amyloid precursors (monoclonal FLC, as defined) are undetected.

Similar relationships between echocardiography parameters and the stage of severity of AL amyloidosis were presented by Kumar et al. in the paper that proposed the current prognostic criteria. [12]. In this study, the median values of IVS in Stages I–IV were, respectively, 11 vs. 13 vs. 15 vs. 16 mm and, for EF values, 65% vs. 63% vs. 60% vs. 53%. In our study, the median number of plasma cells in bone marrow increased together with the mean concentration of involved λ chains.

Analysis of the correlation between the concentration of κ- and λ-involved chains, echocardiography parameters, and TnI and NT-proBNP concentrations confirmed that there is a significant relationship between the concentration of λ-involved chains and the degree of cardiomyocyte injury and myocardial dysfunction. In the group with cardiac involvement and amyloid formed of monoclonal κ chains, similar relationships did not occur. There was no correlation between the concentration of κ chains involved in the process of amyloid formation accumulated in the myocardium and the parameters of echocardiography and TnI concentration. A statistically significant correlation occurred in this group only between the concentration of κ chains involved and the NT-proBNP concentration. Contrary to other results, the clinical studies of Rappezzi et al. showed no correlation between the number of amyloid fibrils present in the myocardium and the degree of its damage and, thus, the average length of survival of patients [23]. On the other hand, Palladini et al. confirmed that the reduction of circulating monoclonal chains in patients with AL amyloidosis resulted in a decrease in NT-proBNP concentration and prolonged overall survival, regardless of the size of amyloid deposits in the heart [24]. Based on clinical observations, it has been hypothesized that monoclonal light chains may have a direct toxic effect on myocardial cells in patients with primary amyloidosis. One of the first studies confirming the direct cardiotoxic effect of light chains of immunoglobulins was conducted by Liao et al. in 2001. Brenner et al. showed that even low levels of sFLC in patients with AL amyloidosis may induce an increase in oxidative stress in isolated cardiomyocytes in vitro, for example, by increasing the amount of reactive oxygen species (ROS) and the imbalance between redox reactions and oxidation. This results in impaired contractility and relaxation of myocardial cells. What should be noted is that these changes were independent of the amounts of fiber and amyloid deposits; they resulted only from the interaction between free “amyloidogenic” FLC and cardiomyocytes [25]. Shi et al. and Sikkink et al. yielded more information about the pathomechanism of oxidative stress caused by FLC itself. The studies showed that FLC isolated from patients with AL amyloidosis with cardiac involvement exacerbated oxidative stress, dysfunction, and apoptosis of cardiomyocytes by activation of MAPK kinase p38 [26,27]. The authors’ own study showed that in the group with myocardial involvement, where the amyloid is formed by the involved λ chains, the only parameter that demonstrated an independent influence on the concentration of TnI was the concentration of λ free chains. However, within the same group, the concentration of NT-proBNP was independently influenced not only by the concentration of λ free chains but also by the eGFR value and the concentration of uninvolved polyclonal κ FLC. On the other hand, multivariable linear regression analysis of the group with myocardial involvement, where the amyloid is formed by monoclonal κ chains, confirmed that the increase in the concentration of involved κ chains did not show an independent, significant influence on both TnI and NT-proBNP concentrations. Interestingly, in the study group, a significant, independent influence of the concentration of uninvolved λ chains on the concentration of NT-proBNP was observed. It was proven in the author’s research that the function of the myocardium in the group of patients with AL amyloidosis is influenced not only by the chains involved in the amyloid formation process but also by the uninvolved light chains. These additional preliminary conclusions should be the subject of further research. The ability to assess the condition of the myocardium using laboratory tests (which, unlike invasive organ biopsies, are safe for the patient, more easily available, with relatively quick results) is undoubtedly important. Evaluation of the heart function “in real-time” is important not only during the diagnosis but, above all, during the monitoring of the applied treatment. One of the main goals of this work was to try to select the type of chain involved in the cancer process, the presence of which is associated with more severe damage to the myocardium and, thus, with a potentially worse prognosis. Only in relation to multiply myeloma, the presence of the monoclonal λ chain has been shown to be an unfavorable prognostic factor [3]. In the present study, a multivariate linear regression analysis showed that in the group with λ chains, the myocardial involvement amount of λ chains had an independent effect on the concentrations of TnI and NT-proBNP. The analysis of the group with myocardial involvement by amyloids formed from monoclonal κ chains confirmed that the increase in the κ-chains involved did not show a significant independent effect on both TnI concentration and NT-pro BNP concentration.

## 5. Conclusions

The above data indicate that the presence of monoclonal λ chains in patients with AL amyloidosis may be associated with more severe damage to cardiomyocytes and dysfunction of the myocardium. To state with finality that monoclonal chain λ is a disadvantageous factor in patients with primary amyloidosis would require further research, preferably multicenter, to assess the dependence of the type of chain involved on the overall survival of patients.

## Figures and Tables

**Table 1:** Criteria for organ * involvement in systemic immunoglobulin light chain amyloidosis (AL amyloidosis) according to Gertz et al. [13,14].

Organ	Criteria for Organ Involvement
Heart	Echocardiographic examination: cardiac wall thickness >12 mm, excluding any other cardiological causes
Kidney	Daily urine collection: proteinuria >0.5 g/day (predominantly albuminuria)
Liver	Total liver size >15 cm, excluding accompanying heart failure, or alkaline phosphatase activity >1.5 upper reference range
Autonomic nervous system	Abnormal gastric peristalsis, micturition disorders, or pseudoconstipation (excluding other organic causes)
Peripheral nervous system	Clinical symptoms: sensorimotor, symmetrical, peripheral neuropathy of the lower extremities
Gastrointestinal tract	Direct biopsy confirmation of the presence of amyloid deposits in conjunction with clinical symptoms
Lung	Biopsy confirmation of the presence of amyloids in conjunction with clinical symptoms; X-ray shows interstitial lesions in the lungs
Soft tissue	Tongue enlargement, intermittent claudication due to the presence of amyloids in the vessels, arthropathy, carpal tunnel syndrome, biopsy, myopathy or lymphadenopathy, skin lesions

* Other organs available for histological verification and confirmation of amyloidosis: abdominal fat, gingiva, rectum, salivary glands.

**Table 2:** Characteristics of the study group.

Study Group 57 (80%) Primary Systemic Light Chain Amyloidosis 10 (14%) Primary Systemic Light Chain Amyloidosis Accompanying Myeloma 4 (6%) Localized Primary Light Chain Amyloidosis
Amyloid structure
43 (61%) λ chains
28 (39%) ĸ chains
Amyloid location by number of sites
22 (31%) one organ
28 (40%) two organs
18 (25%) three or more organs
Amyloid location in organs
51 (72%) heart
41 (58%) kidneys
20 (28%) gastrointestinal tract
10 (14%) liver
3 (4%) bone marrow or in adipose tissue only
14 (20%) others (bronchi, lungs, peripheral nervous system, nasopharynx, tongue, skin)

**Table 3:** Laboratory and clinical findings in enrolled subjects. The *p*-value concerns differences between groups of patients with λ and κ amyloid-forming light chains

Involved Chain	All Me (Q25–75)	Λ Me (Q25–75)	Κ Me (Q25–75)	*p-*Value
*n*	71	43	28	
Age (years)	57 (54–65)	60 (56–64)	55 (51–67)	0.003
Plasmocytes (%)	11 (6–15)	10 (5–15)	15 (6–15)	0.084
TnI (ng/mL)	0.019 (0.000–0.072)	0.030 (0.002–0.100)	0.010 (0.000–0.045)	0.002
NT-proBNP (pg/mL)	1958 (362–7393)	2705 (731–8170)	657 (286–6247)	<0.001
LV (mm)	45 (41–50)	45 (40–51)	45 (40–50)	0.759
IVS (mm)	13 (11–14)	13 (11–14)	12 (11–14)	0.309
PW (mm)	12 (11–14)	13 (11–15)	12 (10–14)	0.246
RV (mm)	30 (27–33)	29 (26–35)	30 (28–32)	0.589
EF (%)	60 (55–65)	60 (55–64)	61 (55–65)	0.203
κ (mg/L)	28.7 (16.0–70.2)	22.5 (15.3–45.5)	41.7 (21.1–103.5)	<0.001
λ (mg/L)	24.8 (15.1–63.9)	42.6 (21.4–99.2)	16.7 (11.8- 24.8)	<0.001
dFLC (mg/L)	12.2 (−0.3–50.7)	4.3 (−6.8–48.1)	17.3 (5.2–51.8)	<0.001
κ/λ	1.21 (0.62–1.94)	0.80 (0.32–1.30)	1.76 (1.29–2.80)	<0.001

TnI-troponin I, NT pro-BNP-N-terminal pro b-type natriuretic peptide, LV-left ventricular end-diastolic dimension, IVS-diastolic dimension of the interventricular septum, PW-thickness of the posterior wall of the left ventricle in diastole, RV-right-ventricular end-diastolic dimension, EF-ejection fraction of the left ventricle, dFLC-difference between the concentration of involved and uninvolved free light chain.

**Table 4:** Comparative analysis of study groups divided based on free chain concentration ratio κ/λ: <0.26, >1.65, and 0.26–1.65.

κ/λ	<0.26 Me (Q25–75)	>1.65 Me (Q25–75)	0.26–1.65 Me (Q25–75)	*p-*Value
*n*	51	146	259	
Age (years)	58 (49–65)	59 (54–63)	56 (51–64)	0.667
Plasmocytes (%)	20 (12–25)	15 (7–15)	10 (5–15)	<0.001
TnI (ng/mL)	0.092 (0.036–0.200)	0.006 (0.000–0.030)	0.002 (0.000–0.039)	<0.001
NT-proBNP (pg/mL)	0.092 (0.036–0.200)	0.006 (0.000–0.030)	0.002 (0.000–0.039)	<0.001
κ (mg/L)	14.5 (10.2–18.3)	76.2 (39.9–148.6)	21.9 (13.6–40.3)	<0.001
λ (mg/L)	188.7 (112.1–280.5)	21.2 (15.1–30.3)	23.1 (13.9–59.9)	<0.001
dFLC	160.2 (92.3–270.0)	23.0 (−13.4–79.3)	3.4 (−1.2–18.6)	<0.001
κ/λ	0.10 (0.04–0.15)	2.47 (1.98–5.08)	0.98 (0.64–1.30)	<0.001
LV (mm)	44 (40–51)	47 (44–52)	46 (43–48)	0.338
IVS (mm)	14 (13–14)	13 (12–13)	12 (11–14)	0.028
PW (mm)	13 (12–15)	13 (12–14)	12 (10–14)	0.158
RV (mmm)	31 (27–33)	31 (28–32)	26 (25–30)	0.063
EF (%)	59 (41–60)	60 (55–63)	60 (55–65)	0.318

**Table 5:** Comparative analysis of study groups with involved and uninvolved free light chains—dFLC <180 and ≥180 mg/L.

dFLC [mg/L]	≥180 Me (Q25–75)	<180 Me (Q25–75)	*p-*Value
Age (years)	59 (50–66)	56 (52–64)	0.425
Plasmocytes (%)	11.5 (8.0–20.0)	12.0 (6.0–15.0)	0.083
TnI (ng/mL)	0.079 (0.035–0.297)	0.003 (0.000–0.039)	<0.001
NT-proBNP (pg/mL)	4425 (724–10577)	1602 (337–4466)	0.014
κ (mg/L)	36.1 (11.8–613.3)	28.9 (16.3–63.7)	0.153
λ (mg/L)	219.4 (10.7–312.8)	24.4 (15.5–59.9)	0.007
κ “i”	616.3 (520.8–775.3)	36.3 (18.7–80.2)	<0.001
λ “i”	289.3 (232.3–617.3)	36.5 (20.4–73.8)	<0.001
κ “ui”	12.2 (10.3–16.9)	25.0 (16.1–46.8)	<0.001
λ “ui”	9.2 (8.1–14.9)	17.8 (12.7–25.5)	<0.001
dFLC (mg/L)	538.8 (236.7–682.4)	8.4 (−1.5–35.4)	<0.001
κ/λ	0.095 (0.04–53.5)	1.23 (0.75–1.90)	0.224
LV (mm)	45 (43–51)	46 (41–51)	0.849
IVS (mm)	14.0 (13–16)	12 (11–13)	0.019
PW (mm)	14 (13–16)	12 (11–14)	0.020
RV (mm)	32 (30–35)	28 (26–32)	0.070
EF (%)	56 (41–65)	60 (55–64)	0.312

“i”—involved free chain; “ui”—uninvolved free chain.

**Table 6:** Comparative analysis of study groups based on the current Kumar et al. prognosis classification (Stages I–IV).

Stage	I Me (Q25–75)	II Me (Q25–75)	III Me (Q25–75)	IV Me (Q25–75)	*p*-Value
Age (years)	56 (49–60)	56 (55–64)	61 (44–65)	57 (53–61)	0.382
lasmocytes (%)	10 (6–15)	12 (5–20)	12 (6–25)	15 (12–20)	0.050
TnI (ng/mL)	0.000	0.034 (0.009–0.043)	0.170 (0.086–0.434)	0.249 (0.120–0.734)	<0.001
NT-proBNP (pg/mL)	313 (98–543)	2960 (1986–7820)	7401 (2985–10500)	10931 (7849–13773)	<0.001
κ (mg/L)	18.1 (11.7–39.3)	48.2 (18.5–130.2)	25.5 (17.2–42.9)	11.3 (9.8–16.7)	<0.001
λ (mg/L)	17.3 (13.1–21.9)	47.3 (24.3–86.9)	65.3 (44.2–116.0)	363.2 (230.1–617.3)	<0.001
dFLC (mg/L)	3.39 (0.74–23.74)	36.69 (1.0–104.81)	49.41 (31.31–100.31)	349.50 (211.36–606.34)	<0.001
κ/λ	1.18 (0.86–1.94)	1.17 (0.40–2.66)	0.48 (0.26–1.21)	0.04 (0.02–0.07)	<0.001
κ “i” (mg/L)	33.6 (11.2–49.5)	130.4 (96.0–182.5)	26.7 (21.3–140.3)	-	<0.001
λ “ui” (mg/L)	16.4 (11.3–20.5)	22.8 (16.0–46.2)	22.1 (20.3–63.3)	-	0.003
κ “ui” (mg/L)	16.7 (12.1–23.7)	27.4 (16.8–77.3)	21.6 (12.6–42.9)	11.3 (9.8–16.7)	0.002
λ “i”	18.8 (13.8–26.8)	63.8 (37.5–105.3)	95.2 (65.2–162.4)	363.2 (230.1–617.3)	<0.001
LV (mm)	44 (44–47)	45 (40–50)	37 (36–47)	48 (41–52)	0.556
IVS (mm)	12 (11–12)	13 (11–14)	15 (11–17)	14 (13–17)	0.015
PW (mm)	12 (10–12)	12 (11–14)	14 (13–14)	15 (13–16)	0.010
RV (mm)	30 (25–32)	27 (27–28)	26 (26–27)	33 (32–35)	0.038
EF (%)	60 (55–63)	60 (59–65)	59 (50–60)	39 (28–56)	0.025

“i”—involved free light chains, “ui”—univolved free light chains.

**Table 7:** Comparative analysis depending on the diagnosis.

Diagnosis	AL Amyloidosis	AL Amyloidosis Associated with Myeloma	Localized AL Amyloidosis	
	Me (Q25–75)	Me (Q25–75)	Me (Q25–75)	*p*-Value
Age (years)	56 (54–64)	63 (41–65)	70 (44–70)	<0.001
Plasmocytes (%)	10 (5–15)	15 (15–25)	6 (6–8)	<0.001
TnI (ng/mL)	0.021 (0.000–0.076)	0.039 (0.007–0.083)	0.000 (0.000–0.000)	<0.001
NT-proBNP (pg/mL)	1932 (382–7401)	4499 (1958–8711)	106 (88–271)	<0.001
κ (mg/L)	29.83 (16.18–75.70)	29.27 (18.46–65.65)	12.62 (9.95–24.55)	0.009
λ (mg/L)	23.83 (15.10–61.77)	47.34 (23.39–112.06)	13.98 (12.26–15.13)	<0.001
κ “i”	43.95 (21.78–103.00)	562.40 (429.60–787.00)	NA	<0.001
λ “i”	39.51 (21.35–95.06)	53.10 (26.17–113.13)	NA	0.397
κ “ui”	22.02 (14.62–45.73)	25.32 (17.46–48.16)	NA	0.204
λ “ui”	18.26 (11.39–25.51)	15.50 (13.40–47.34)	NA	0.132
dFLC (mg/L)	13.85 (0.22–48.73)	8.42 (−2.40–102.50)	NA	0.008
κ/λ	1.32 (0.75–2.02)	0.87 (0.31–1.34)	0.94 (0.80–1.64)	<0.001
LV (mm)	46 (43–50)	42 (40–46)	50 (43–55)	0.087
IVS (mm)	13 (11–14)	14 (13–17)	10 (8–11)	0.162
PW (mm)	12 (11–14)	15 (12–15)	9 (8–10)	0.029
RV (mm)	30 (27–35)	28 (27–32)	28 (26–30)	0.319
EF (%)	60 (56–65)	60 (55–64)	55 (55–60)	0.517

“i”—involved free chain; “ui”—uninvolved free chain. NA—not applicable.

**Table 8:** Multivariable linear regression model of TnI and NT pro-BNP concentrations in the group with involvement of the myocardium by amyloids formed from monoclonal λ chains.

Variable	Effect	SE	95% CI	*p*	R^2^	Beta
TnI				0.003	0.130	
λ	0.0005	0.0001	28.01 to 54.59	0.003		0.360
NT pro-BNP				<0.001	0.340	
λ	41.302	6.708	28.01 to 54.59	<0.001		0.475
eGFR	–63.396	32.849	–128.48 to 1.69	0.042		–0.171
κ	114.051	37.495	39.76 to 188.34	0.003		0.270

CI = confidence interval; SE = standard error.

**Table 9:** Multivariable linear regression analysis for NT-proBNP concentration in the group with myocardial involvement where the amyloid is formed by monoclonal κ chains.

Variable	Effect	SE	95% CI	*p*	R^2^	Beta
NT pro-BNP				<0.001	0.370	
λ	204.254	31.603	141.24 to 267.269	<0.001		0.609

CI = confidence interval; SE = standard error.

**Table 10:** Correlation coefficients (Rs) for κ and λ light chains, dFLC values, and κ/λ ratio with myocardium involvement parameters and echo test in the group with involvement of the myocardium by amyloids formed from monoclonal λ and κ chains.

	Monoclonal λ Chains				Monoclonal κ Chains			
	κ	λ	dFLC	κ/λ	κ	λ	dFLC	κ/λ
**LV**	0.280	−0.238	−0.438 *	0.431 *	−0.051	−0.018	−0.113	−0.188
**IVS**	−0.358	0.496 *	0.434 *	−0.465 *	0.111	0.211	−0.193	−0.247
**PW**	−0.060	0.323	0.092	−0.132	0.016	0.333	−0.202	−0.244
**EF**	0.297	−0.107	−0.077	0.130	−0.052	0.196	−0.063	−0.128
**TnI**	−0.278	0.688 *	0.669 *	−0.694 *	−0.215	0.215	−0.269	−0.398 *
**NTpro-BNP**	0.263 *	0.449 *	0.053	−0.080	0.324 *	0.695 *	−0.027	−0.314 *

* *p* < 0.05.

## Data Availability

The data presented in this study are available on request from the corresponding author. The data are not publicly available due to ethical reasons.

## References

[1] Dispenzieri A., Gertz M.A., Buadi F. (2012). What do I need to know about immunoglobulin light chain (AL) amyloidosis?. Blood Rev..

[2] Charliński G., Juryszczyn A., Jędrzejczak W.W. (2014). Light chain amyloidosis–clinical symptoms, updated diagnosis, and treatment. Med. Rev..

[3] Dmoszyńska A., Walter-Croneck A., Pieńkowska-Grela B. (2016). Recommendations of Polish Myeloma Group concerning diagnosis and therapy of multiple myeloma and other plasmacytic dyscrasias for 2017. Acta Haematol. Pol..

[4] Charliński G., Jędrzejczak W.W., Dmoszyńska A., Giannopoulous K. (2015). Primary systemic light-chain amyloidosis: Diagnosis and treatment. Multiple Myeloma and other Plasma Cell Dyscrasias.

[5] Kumar S., Dispenzieri A., Lacy M.Q., Hayman S.R., Buadi F.K., Colby C., Laumann K., Zeldenrust S.R., Leung N., Dingli D. (2012). Revised prognostic staging system for light chain amyloidosis incorporating cardiac biomarkers and serum free light chain measurements. J. Clin. Oncol..

[6] Desport E., Bridoux F., Sirac C., Delbes S., Bender S., Fernandez B., Quellard N., Lacombe C., Goujon J.-M., Lavergne D. (2012). AL Amyloidosis. Orphanet J. Rare Dis..

[7] Selvanayagam J.B., Hawkins P.N., Paul B., Myerson S.G., Neubauer S. (2007). Evaluation and management of the cardiac amyloidosis. J. Am. Coll. Cardiol..

[8] Jurczyszyn A., Engel A., Rajzer M., Czepiel J., Mazur G. (2014). Progress in the diagnosis and treatment of cardiac amyloidosis. Med. Rev..

[9] Guan J., Mishra S., Falk R.H., Liao R. (2012). Current perspectives on cardiac amyloidosis. Am. J. Physiol. Heart Circ. Physiol..

[10] Shah K.B., Inoue Y., Mehra M.R. (2006). Amyloidosis and the heart: A comprehensive review. Arch. Intern. Med..

[11] Falk R.H. (2005). Diagnosis and management of the cardiac amyloidoses. Circulation.

[12] Kumar S., Dispenzieri A., Katzmann J.A. (2010). Serum immunoglobulin free light chain measurement in AL amyloidosis: Prognostic value and correlations with clinical features. Blood.

[13] Gertz M.A., Comenzo R., Falk R.H., Fermand J.P., Hazenberg B.P., Hawkins P.N., Merlini G., Moreau P., Ronco P., Sanchorawala V. (2005). Definition of organ involvement and treatment response in immunoglobulin light chain amyloidosis (AL): A consensus opinion from the 10th International Symposium on Amyloid and Amyloidosis, Tours, France, 18–22 April 2004. Am. J. Hematol..

[14] Gertz M.A., Merlini G. (2010). Definition of organ involvement and response to treatment in AL amyloidosis: An updated consensus opinion. Amyloid.

[15] Bochtler T., Hegenbart U., Heiss C., Benner A., Cremer F., Volkmann M., Ludwig J., Perz J.B., Ho A.D., Goldschmidt H. (2008). Evaluation of the serum-free light chain test in untreated patients with AL amyloidosis. Haematologica.

[16] Snozek C.L., Katzmann J.A., Kyle R.A., Dispenzieri A., Larson D.R., Therneau T.M., Melton L.J., Kumar S., Greipp P.R., Clark R.J. (2008). Prognostic value of the serum free light chain ratio in newly diagnosed myeloma: Proposed incorporation into the international staging system. Leukemia.

[17] Kyle R.A., Gertz M.A. (1995). Clinical and laboratoty features in 474 cases. Semin. Hematol..

[18] Merlini G., Sanchorawala V., Zonder J.A., Kukreti V., Schonland O.S., Jaccard A., Dispenzieri A., Cohen A.D., Berg R.D., Liu G. (2012). MLN9708, a novel, investigational oral proteasome inhibitor, in patients with relapsed or refractory light chain amyloidosis (AL): Results of a phase 1 study. Blood.

[19] Lee M.H., Lee S.P., Kim Y.J., Sohn D.W. (2013). Incidence, Diagnosis and Prognosis of Cardiac Amyloidosis. Korean Circ. J..

[20] Huang X., Wang Q., Jiang S., Chen W., Zeng C., Liu Z. (2015). The clinical features and outcomes of systemic AL amyloidosis: A cohort of 231 Chinese patients. Clin. Kidney J..

[21] Dispenzieri A., Gertz M.A., Kumar S.K., Lacy M.Q., Kyle A.R., Saenger A.K., Grogan M., Zeldenrust S.R., Hayman S.R., Buadi F. (2014). High sensitivity cardiac troponin T in patients with immunoglobulin light chain amyloidosis. Heart.

[22] Dispenzieri A., Lacy M.Q., Katzmann J.A., Rajkumar S.V., Abraham R.S., Hayman S.R., Kumar S.K., Clark R., Kyle R.A., Litzow M.R. (2006). Absolute values of immunoglobulin free light chains are prognostic in patients with primary systemic amyloidosis undergoing peripheral blood stem cell transplantation. Blood.

[23] Rapezzi C., Merlini G., Quarta C.C., Riva L., Longhi S., Leone O., Salvi F., Ciliberti P., Pastorelli F., Biagini E. (2009). Systemic cardiac amyloidosis: Disease profiles and clinical courses of the 3 main types. Circulation.

[24] Palladini G., Lavatelli F., Russo P., Perlini S., Perfetti V., Bosoni T., Obici L., Bradwell A.R., D’Eril G.M., Fogari R. (2006). Circulating amyloidogenic free light chains and serum N-terminal natriuretic peptide type B decrease simultaneously in association with improvement of survival in AL. Blood.

[25] Brenner D.A., Jain M., Pimentel D.R., Wang B., Connors L.H., Skinner M., Apstein C.S., Liao R. (2004). Human amyloidogenic light chains directly impair cardiomyocyte function through an increase in cellular oxidant stress. Circ. Res..

[26] Shi J., Guan J., Jiang B., Brenner D.A., Del Monte F., Ward J.E., Connors L.H., Sawyer D.B., Semigran M.J., MacGillivray T.E. (2010). Amyloidogenic light chains induce cardiomyocyte contractile dysfunction and apoptosis via a non-canonical p38alpha MAPK pathway. Proc. Natl. Acad. Sci. USA.

[27] Sikkink L.A., Ramirez-Alvarado M. (2010). Cytotoxicity of amyloidogenic immunoglobulin light chain in cell culture. Cell Death Dis..

